# Biliary atresia combined Wilson disease identified by whole exome sequencing in Vietnamese patient with severe liver failure

**DOI:** 10.1097/MD.0000000000028547

**Published:** 2022-01-14

**Authors:** Nguyen Pham Anh Hoa, Nguyen Thi Kim Lien, Nguyen Van Tung, Nguyen Ngoc Lan, Nguyen Thi Phuong Mai, Nguyen Thi Mai Huong, Hoang Ngoc Thach, Nguyen Huy Hoang

**Affiliations:** a Hepatology Department, Vietnam National Hospital of Pediatrics, Ministry of Health, 18/879 La Thanh Str., Dongda, Hanoi, Vietnam; b Institute of Genome Research, Vietnam Academy of Science and Technology, 18 - Hoang Quoc Viet Str., Caugiay, Hanoi, Vietnam; c Graduate University of Science and Technology, Vietnam Academy of Science and Technology, Vietnam; d Human Genetics Department, Vietnam National Hospital of Pediatrics, Ministry of Health, 18/879 La Thanh Str., Dongda, Hanoi, Vietnam; e Pathology Department, Vietnam National Hospital of Pediatrics, Ministry of Health, 18/879 La Thanh Str., Dongda, Hanoi, Vietnam.

**Keywords:** *ABCB11*, *ATP7B*, biliary atresia, *KRT18*, PFIC2, Wilson disease

## Abstract

**Rationale:**

Hepatobiliary diseases such as biliary atresia (BA), Wilson disease, and progressive familial intrahepatic cholestasis are common causes of morbidity and mortality in young children. Affected patients progress rapidly to end-stage cirrhosis and require liver transplantation or die. Mutations in many genes have been identified to play an important role in the pathogenesis of hepatobiliary diseases.

**Patient concerns and diagnosis:**

In this study, we identified mutations in an 8-year-old girl who had severe liver failure. The patient was first diagnosed with BA at 2.5 months of age and has undergone Kasai surgery to connect the umbilical cord and jejunum. After that, the patient suddenly had unusual developments with symptoms of jaundice, acute liver failure with hemolysis. She was tested and diagnosed with Wilson disease.

**Interventions and outcomes:**

She was treated according to the regimen for a patient with Wilson disease but had abnormal progress leading to severe liver failure. Genetic analysis was performed by whole exome sequencing and Sanger sequencing methods. The genetic analysis revealed that the patient had a homozygous mutation (p.Gly17Glyfs77∗) in the *KRT18* gene, a double heterozygous mutation (p.Ser105∗ and p.Pro992Leu) in the *ATP7B* gene, and a homozygous variant (p.Val444Ala) in the *ABCB11* gene. *In silico* prediction of mutations indicated that these mutations are the cause of the severe liver failure in the patient.

**Lesson:**

This is a rare clinical case of a BA patient combined with Wilson disease. Our results suggested that whole exome sequencing is an effective diagnostic tool and emphasizes the importance of early diagnosis and appropriate management to save lives and prevent serious complications in the patient.

## 1 Introduction

In infancy, the infant's liver is often not physiologically complete therefore the liver is very susceptible to damage in the development. Hepatobiliary diseases often present and threaten the infant's life leading to a high mortality rate if not treated promptly in infancy. Hepatobiliary diseases affect seriously the health of infants such as biliary atresia (BA), Wilson disease, and progressive familial intrahepatic cholestasis diseases. In many cases, these diseases do not have obvious clinical manifestations, so the diagnosis and treatment for the patient face many difficulties.^[1]^ The challenge is the differential diagnosis of diseases with similar clinical manifestations.^[2]^ Advances in deoxyribose nucleotide acid (DNA) technology, such as whole genome sequencing and whole exome sequencing (WES), have improved our understanding of the pathogenesis of these diseases. With the help of these methods, many mutations in genes associated with hepatobiliary diseases have been identified in recent years.

BA is a disease characterized by progressive inflammation, fibrosis, destruction of the bile ducts, leading to fibrosis and obstruction of the biliary. It is a rare disease with a frequency of about 1/18,000 live births in Europe, but higher in Asia with a prevalence of 1/5000.^[3]^ Clinical manifestations are characterized by the rise of conjugated or direct bilirubin, dark urine, fecal discoloration, hepatosplenomegaly, and progressive liver failure. If untreated, the patients will develop rapidly progress fibrosis, leading to end-stage liver disease and death within the first 2 years of life.^[3]^ The pathogenesis of BA is not clearly understood. Kasai surgery is a temporary fix but is the optimal treatment for patients with BA in the early stages. However, 70% to 80% of patients with successful Kasai surgery still have biliary fibrosis leading to cirrhosis and need a liver transplant. Mutations in several genes have been identified as the cause and/or susceptibility factor of the disease. In recent years, WES has been used to identify genetic variants associated with Mendelian disorders in children including BA.

Wilson disease is an autosomal recessive disorder characterized by the accumulation of copper in the liver, brain, and cornea. The disease has an onset in childhood and result in significant neurological impairment or require lifelong treatment. Another serious consequence of the disease is the development of liver damage and acute liver failure leading to transplantation. A significantly higher frequency of Wilson disease in East Asian populations than that in other populations, it is ranging from 1:30,000 to 1:50,000.^[4]^ The disorder is caused by mutations in the *ATP7B* gene, encoding a P-type copper transporting adenosine triphosphatase localized on chromosome 13 at 13q14.3.^[4]^ The identification of mutations in the *ATP7B* gene is one of the useful tools for diagnosis and treatment orientation for patients with Wilson disease.

Progressive familial intrahepatic cholestasis type 2 belongs to the group of cholestatic jaundice caused by abnormalities in bile secretion, cholestatic jaundice, and severe liver damage.^[1,2]^ The incidence of children is 1:50,000 and 1:100,000.^[1]^ The clinical symptoms of progressive familial intrahepatic cholestasis type 2 such as cholestatic jaundice, discolored stools, and dark yellow urine are difficult to distinguish from other causes of cholestatic jaundice. The diagnosis is confirmed by mutations in the *ABCB11* gene (2q24), the gene encoding bile salt transport protein.^[1]^ These mutations cause the impaired secretion of bile acids synthesized at the liver resulting in the accumulation of bile salts in hepatocytes and severe liver damage within the first year.^[1]^

In this study, we identified gene mutations in an 8-year-old girl who had a severe liver failure by WES and Sanger sequencing methods. To predict the affection of mutations on pathogenesis, we used prediction software including SIFT, PolyPhen2, Mutation Taster, PROVEAN, and SNP&GO.

## 2 Case presentation

**Figure 1:**
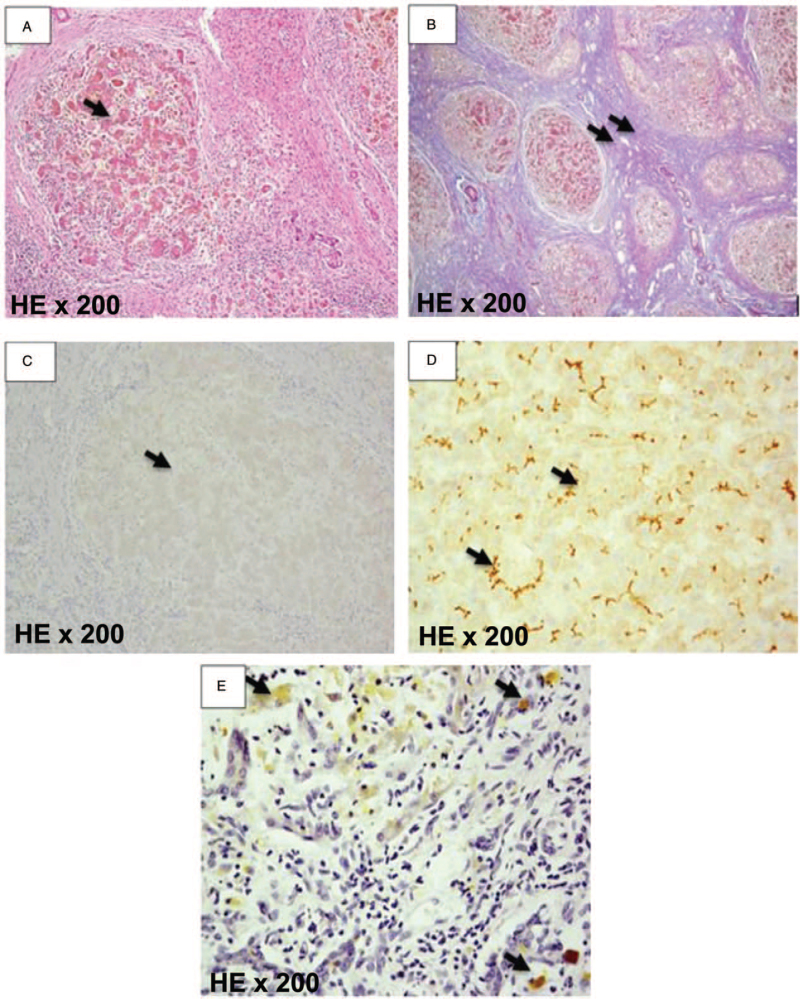
Liver biopsy specimen (H + E, ×200). (A) Liver fibrosis is covered with fibrous tissue and the remaining degenerative liver cells without a central vein. (B) Liver nodules and fibrous septum stained with Trichrome. (C) Liver nodules negative with BSEP, stained by immunohistochemical (IHC). (D) Liver nodules positive with BSEP, stained by IHC. (E) Liver nodules positive with copper, stained by Rhodamine dye. BSEP = bile salt transport protein.

The patient was diagnosed with BA at 2.5 months of age with symptoms of cholestatic jaundice, stained feces, hepatosplenomegaly, small-sized gallbladder ultrasound (12 × 3 mm), no contraction before and after a meal, and TC sign 3.5 mm. The patient has undergone Kasai surgery to connect the umbilical cord and jejunum at The Hepatology Department. She was diagnosed with BA type 4 according to the classification of the Japanese Pediatric Surgery Association. Liver tissue biopsy showed various liver lesions including cholestasis in the liver, inflammatory cells concentrated in the portal space, proliferation of immature bile, and degree III-IV cirrhosis (Fig. 1). Kasai surgery was successful and evaluated to be in the group with a good prognosis after treatment. The patient had no symptoms of jaundice 4 months after surgery and remained stable for the first 3 years of life. When she was 8 years old, the patient suddenly had unusual developments with symptoms of jaundice, acute liver failure with hemolysis. The patient was admitted to the hospital with dark jaundice, dark urine, left lobe of the liver to 4 cm in size, spleen enlarged degree II. These were the main reasons why the patient was indicated additional screening tests for other liver diseases, which focused on groups of diseases leading to acute liver injury accompanied by hemolysis, including Wilson and progressive familial intrahepatic cholestasis diseases. The patient was diagnosed with Wilson disease due to symptoms including acute liver failure with hemolytic, negative Coombs test, and low level of alkaline phosphatase. Subclinical tests such as 24-hour urine, ceruloplasmin, the Kayser-Fleischer ring, and genetic analysis were used to diagnose Wilson disease (Table 1). The result tests showed that she had Kayser-Fleischer ring but negative Coombs test. The histological evaluation of the liver demonstrated the copper accumulation in liver tissue (Fig. 1). She was diagnosed with Wilson disease with a 10-point scale according to the criteria of Leipzig 2001 and treated with Wilson patient regimens and supportive treatments for patients with end-stage liver disease. Drugs used include D penicillamine, zinc acetate, ursodeoxycholic acid, vitamin K, vitamins A, D, E, and vitamin B6 supplements. She also received supportive treatment for liver failure including albumin and fresh plasma compensation, dietary protein restriction, and acid-alkaline balance adjustment. However, the symptoms of hepatic impairment were deteriorating with hepatic coma episodes that occurred consecutively against the background of chronic liver disease with severe hemolytic episodes. She was treated 3 times with plasma filtration due to hepatic coma episodes and elevated blood ammonia level, the patient was determined to have end-stage liver disease (PELD-score of 32 points) and assigned a liver transplant.

**Table 1:** Subclinical symptoms of the patient at 8 years of age.

Tests	Normal	Patient
Hemoglobin (g/dL)	11.0–14.0	9.1
WBC (×10^9^/L)	4.3–10.8	5.9
Platelet (thrombocytes) (×10^9^/L)	150.0–300.0	36.0
Total bilirubin (μmol/L)	3.4–17.0	716.0
Direct bilirubin (μmol/L)	0.5–6.8	328.0
AST (U/L)	<40.0	124.0
ALT (U/L)	<40.0	138.0
GGT (IU/L)	<40.0	86.5
PT%	>70.0	32.7
INR	0.8–1.0	3.1
Albumin (g/L)	35.0–50.0	28.0
Protein (g/L)	60.0–80.0	52.0
Coombs	Negative	Negative
Kayser-Fleischer ring	Negative	Positive
Urea (μmol/L)	3.3–6.6	1.6
Creatinine (μmol/L)	40.0–85.0	46.0
Ammonia (μmol/L)	<50.0	210.0
Ceruloplasmin (g/L)	0.2–0.6	0.064
24 h urinary copper (mg/dL)	<0.1	0.5
Serum copper (mg/dL)	12.0–28.0	12.0

ALT = alanin amino transferase, AST = aspartate amino transferase, GGT = gamma glutamyl transferase, INR = international normalized ratio, PT = prothrombin time, WBC = white blood cell.

### 2.1 Genetic analysis

Genomic DNA was isolated from a peripheral blood sample using a Qiagen DNA blood mini kit (QIAamp DNA Blood Mini preparation kits, German) following the manufacturer's guidelines. The DNA sample was used to build the libraries with the Agilent SureSelect Target Enrichment kit (Illumina, CA). After that, WES sequencing was performed using the SureSelect V7-Post kit (Illumina, CA) on the Illumina sequencing machine (Illumina, CA, USA). The data were mapped with the human genome reference (GRCh38) and identified mutations using BWA version 0.7.12 (http://bio-bwa.sourceforge.net/bwa.shtml), Picard version 1.130 (http://broadinstitute.github.io/picard/), GATK version 3.4.0 (https://www.broadinstitute.org/gatk/), SnpEff version 4.1g (http://snpeff.sourceforge.net/SnpEff.html) software for identification, annotation, and prediction of the effects of variants. Candidate variants associated with the disease were filtered according to minor allele frequency <1% in multiple databases including the 1000 Genome Project (https://www.internationalgenome.org/) and gnomAD (https://gnomad.broadinstitute.org/). To validate mutations, the exons and exon-intron boundaries of related genes were amplified and analyzed by direct sequencing. PCR reactions were performed according to previous publications to amplify the exons carrying the mutations in the *ATP7B* and *ABCB11* genes.^[5,6]^ For mutation in the *KRT18* gene, we used primer pairs of forward primer 5′-CAGCATGAGCTTCACCACTC-3′ and reverse primer 5′-GGGAGTTGAGGTTCCCTCCTA-3′ to amplify and sequence. PCR products were purified with the Qiagen PCR Purification kit (QIAGEN, Hilden, Germany) and sequenced on ABI PRISM 3500 Genetic Analyser system (Applied Biosystems, USA). Sequencing data were analyzed and compared with sequences of the *KRT18*, *ATP7B,* and *ABCB11* genes in Ensembl (ENSG00000111057, ENSG00000123191, and ENSG00000073734, respectively) by BioEdit software version 7.0.9.0.

### 2.2 *In silico* analysis

To predict the influence of mutations on the pathogenesis, we were used prediction software including SIFT (https://sift.bii.a-star.edu.sg/), PolyPhen2 (http://genetics.bwh.harvard.edu/pph2/), Mutation Taster (http://www.mutationtaster.org/), PROVEAN (http://provean.jcvi.org/seq_submit.php), and SNP&GO (https://snps.biofold.org/snps-and-go/snps-and-go.html).

## 3 Discussion

BA is a rare type of hepatobiliary disease but is common in Asian children. In this case, the clinical of the patient at 2.5 months of age was consistent with the BA. Up to now, Kasai surgery is considered an effective treatment method for patients with BA. After surgery, she was regularly monitored and assessed to be in the group with a good prognosis, good bile fluid status, and yellow stools. However, the patient's clinical progression was inconsistent with that of a patient with postoperative BA, she was screened for possible causes of acute hemolytic liver injury. The result tests showed that she had Kayser-Fleischer ring but negative Coombs test. The histological evaluation of the liver demonstrated the copper accumulation in liver tissue and was diagnosed as Wilson disease.

**Figure 2:**
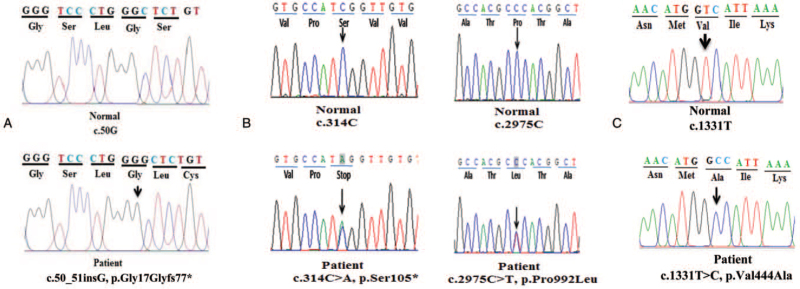
Mutation analysis in the patient. The patient carried a homozygous mutation (p.Gly17Glyfs77∗) in the *KRT18* gene (A), a double heterozygous mutation (p.Ser105∗ and p.Pro992Leu) in the *ATP7B* gene (B), and a homozygous variant (p.Val444Ala) in the *ABCB11* gene (C).

Genetic analysis revealed that she carried a homozygous mutation (p.Gly17Glyfs77∗) in the *KRT18* gene (Fig. 2) that has been determined to be related to BA.^[7]^ The p.Gly17Glyfs77∗ mutation is a new mutation that has not been published on the SNP and ClinVar database. This mutation leads to the formation of a truncated protein which function is affected by the Mutation Taster software prediction (Table 2). Truncated protein mutations are considered mutations affecting protein function and have been identified as pathogenic mutations in many genetic diseases.^[8]^ Besides that 2 heterozygous mutations (p.Ser105∗ and p.Pro992Leu) in the *ATP7B* gene were identified in the patient (Fig. 2). The mutations in the *ATP7B* gene have been evaluated as pathogenic mutations in the ClinVar database and previous studies.^[6]^ The patient also carried a homozygous variant (p.Val444Ala) in the *ABCB11* gene (Fig. 2). This variant may have caused an accumulation of bile salts leading to inflammation and fibrosis of the biliary tract.^[9]^ Lesions and fibrosis of the extrahepatic bile ducts are considered the main symptoms of BA and damage to the bile ducts in the liver is thought to result in the progression of the disease.^[3]^ It may be a factor leading to the clinical abnormalities of the patient and making the difficult process of diagnosis and treatment for the patient. The variant (p.Val444Ala) in the *ABCB11* gene was also found to be associated with susceptibility to BA in Vietnamese patients (data not shown). This result suggests this variant may play a role in causing clinical abnormalities in the patient. Based on clinical symptoms, laboratory tests, and genetic analysis, the patient shows a combination of 2 rare hepatobiliary diseases, BA and Wilson disease. The combination of 2 hepatobiliary diseases in the same patient has also been reported in recent studies.^[10–12]^

**Table 2:** The variants detected in the study.

Gene	*KRT18*	*ATP7B*	*ATP7B*	*ABCB11*
Nucleotide change (Zygosity)	c.50_51insG (Hom)	c.314C>A (Het)	c.2975C>T (Het)	c.1331T>C (Hom)
Amino acid change	p.Gly17Glyfs77∗	p.Ser105∗	p.Pro992Leu	p.Val444Ala
gnomAD frequency	0.31975000	0.00002900	0.00003185	0.56990000
SNP ID	Novel	rs753236073	rs201038679	rs2287622
ClinVar	Not reported	Pathogenic	Pathogenic	Benign
Effect prediction score				
SIFT			0.00 (deleterious)	0.59 (tolerated)
PolyPhen 2			1.00 (damage)	0.00 (benign)
Mutation Taster	1.00 (disease)	1.00 (disease)	0.99 (disease)	0.07 (polymorphism)
PROVEAN			−8.93 (deleterious)	0.01 (neutral)
SNP&GO			RI10 (disease)	RI3 (neutral)

Het = heterozygous, Hom = homozygous.

## 4 Conclusion

In this study, we report a case with severe liver failure due to BA combined with Wilson disease and carried a homozygous variant in the *ABCB11* gene. This combination may be the main cause of abnormal and serious complications leading to end-stage cirrhosis in the patient. Our results contribute to a general understanding of the pathogenesis of hepatobiliary diseases. The results suggest that WES is a powerful tool for the definitive diagnosis and emphasizes the importance of early diagnosis and appropriate management are necessary to save lives and prevent serious complications in the patient.

## Acknowledgments

We are grateful to the patient who participated in this study.

## Author contributions

**Conceptualization:** Nguyen Pham Anh Hoa.

**Funding acquisition:** Nguyen Huy Hoang.

**Methodology:** Nguyen Van Tung, Nguyen Ngoc Lan, Nguyen Thi Phuong Mai, Nguyen Thi Mai Huong, Hoang Ngoc Thach.

**Resources:** Hoang Ngoc Thach.

**Software:** Nguyen Van Tung, Nguyen Ngoc Lan, Nguyen Thi Phuong Mai, Nguyen Thi Mai Huong, Hoang Ngoc Thach.

**Validation:** Nguyen Pham Anh Hoa.

**Visualization:** Nguyen Pham Anh Hoa

**Writing – original draft:** Nguyen Pham Anh Hoa, Nguyen Thi Kim Lien.

**Writing – review & editing:** Nguyen Pham Anh Hoa, Nguyen Thi Kim Lien, Nguyen Van Tung, Nguyen Ngoc Lan, Nguyen Thi Phuong Mai, Nguyen Thi Mai Huong, Hoang Ngoc Thach, Nguyen Huy Hoang.
